# Protein domains provide a new layer of information for classifying human variations in rare diseases

**DOI:** 10.3389/fbinf.2023.1127341

**Published:** 2023-02-21

**Authors:** Mélanie Corcuff, Marc Garibal, Jean-Pierre Desvignes, Céline Guien, Coralie Grattepanche, Gwenaëlle Collod-Béroud, Estelle Ménoret, David Salgado, Christophe Béroud

**Affiliations:** ^1^ Aix Marseille University, INSERM, MMG, Bioinformatics & Genetics, Marseille, France; ^2^ Laboratoire de Génétique Médicale, APHM Hôpital d'Enfants de la Timone, Marseille, France

**Keywords:** ACMG guidelines, variant classification, PM1, PM2, BS1, BP8, protein domain

## Abstract

**Introduction:** Using the ACMG-AMP guidelines for the interpretation of sequence variants, it remains difficult to meet the criterion associated with the protein domain, PM1, which is assigned in only about 10% of cases, whereas the criteria related to variant frequency, PM2/BA1/BS1, is reported in 50% of cases. To improve the classification of human missense variants using protein domains information, we developed the DOLPHIN system (https://dolphin.mmg-gbit.eu).

**Methods:** We used Pfam alignments of eukaryotes to define DOLPHIN scores to identify protein domain residues and variants that have a significant impact. In parallel, we enriched gnomAD variants frequencies for each domains’ residue. These were validated using ClinVar data.

**Results:** We applied this method to all potential human transcripts’ variants, resulting in 30.0% being assigned a PM1 label, whereas 33.2% were eligible for a new benign support criterion, BP8. We also showed that DOLPHIN provides an extrapolated frequency for 31.8% of the variants, compared to the original frequency available in gnomAD for 7.6% of them.

**Discussion:** Overall, DOLPHIN allows a simplified use of the PM1 criterion, an expanded application of the PM2/BS1 criteria and the creation of a new BP8 criterion. DOLPHIN could facilitate the classification of amino acid substitutions in protein domains that cover nearly 40% of proteins and represent the sites of most pathogenic variants.

## Introduction

With the availability of high-throughput sequencing technologies, thousands of variants are identified in every patient analyzed. Up to 50% of coding variants correspond to non-synonymous changes, the interpretation of which is now recognized as the most difficult part of the molecular diagnostic procedure. To improve this process and ensure consistent data interpretation, various guidelines have been proposed (Gelb et al., 2018; Shinar et al., 2020; Baumgartner-Parzer et al., 2020) that rely on the collection of pathogenicity evidence gathered from population data, computational and predictive data, functional data, segregation data, and other levels, as illustrated in the ACMG-AMP guidelines (Richards et al., 2008; Richards et al., 2015). Despite their simplicity, inconsistent classifications between laboratories have been reported (Yorczyk et al., 2015; Kim et al., 2019) and are primarily related to the degrees of subjectivity and uncertainty allowed by the ACMG-AMP guidelines. They recommend using 28 criteria during the interpretation process to distinguish: benign, probably benign, of unknown significance (VUS), probably pathogenic and pathogenic variants. However, only a subset of these criteria are available in clinical practice and access to an annotated collection of variants is essential. To provide such a resource, various initiatives have been developed, including ClinVar (Landrum et al., 2016), ClinGen (Savatt et al., 2018), VarSome (Kopanos et al., 2019) and InterVar (Li and Wang, 2017). These collect data from experts and various resources and can provide an interpretation for unreported variants. Nevertheless, this automated process can sometimes generate inappropriate results and data should be viewed with caution.

If we focus on classification evidence, on the one hand, one of the most challenging criteria is PM1 “Located in a mutational hot spot and/or critical and well-established functional domain (e.g., active site of an enzyme) without benign variation”, which was used in about 10% of reported cases (Amendola et al., 2016). To extract this information, automated systems rely primarily on UniProt (The Uniprot Consortium. 2017) and “dbnsfp31a_interpro”, which is a database of domain information from dbNSFP (Liu et al., 2011; Liu et al., 2016) and InterPro (Mitchell et al., 2019) which incorporates information on protein families, domains, and functional sites. Other initiatives have been developed using the Conserved Domain Database (CDD) (Marchler-Bauer et al., 2015), such as the subRVIS score (Gussow et al., 2016), which aims to assess the intolerance of gene subregions to variants. In general, the PM1 criterion is associated with a broad view of the functional regions where mutations cluster. However, it is difficult to use because this clustering is poorly defined and understood, as illustrated by its various interpretations in Vasome (Kopanos et al., 2019) and InterVar (Li and Wang, 2017). It can also be highly biased by the interest of genes in human diseases and thus to the number of variations classified.

On the other hand, the most used evidence is the PM2/BA1/BS1 “Variant absent in population databases or with an allele frequency too high for the disease” reported in about 50% of cases (Amendola et al., 2016). The assumption of this criterion is very simple: if a variant has been reported with a high frequency in the general population, it cannot be a rare pathogenic variant, otherwise the frequency of this disease would be higher; if a variant has never been reported, or with a very low frequency, then it can be a rare pathogenic variant. This information benefits greatly from the large-scale genome/exome sequencing projects and most people collect this information from gnomAD (Koch, 2020) or population-specific databases, such as the ABraOM (Brazilian population) (Naslavsky et al., 2017), TogoVar (Japanese population) (Mitsuhashi et al., 2022) or Greater Middle East Variome (Middle East population) (Scott et al., 2016). Nevertheless, human evolution has not allowed for genome saturation of variants and some of them are very rare in the population due to genetic drift (Bach, 2019). Indeed, the chance appearance of a neutral variant not subject to a selection force will most likely result in its disappearance after several generations if the population is large enough, and only a few will be fixed in the population. Thus, while it is recognized that 50 to 100 *de novo* variants appear each generation in humans, most of these events have been lost during evolution, explaining why not all neutral substitutions are present in our genome. An alternative view is based on the simple assumption that the 50 to 100 *de novo* variants in each of the 7.8 billion living humans should have produced every nucleotide change compatible with life (Shirts et al., 2016). Only the availability of all human genomes would allow us to conclude whether either of these views is correct or whether the truth lies somewhere in between.

How can the evidence for PM1 and PM2/BA1/BS1 be linked? We expect this to be the case simply because the information contained in the protein domains can be used to shed light on both. We hypothesized that alignment of protein domains will identify key residues involved in the structure or function of these domains and identify variations that strongly affect these properties, and that the frequency of substitutions at each position in all homologous domains will enrich our interpretation of the frequency of a variant in the population.

To extract such information from protein domains, we developed the DOLPHIN “Domain Logo Protein for Human Information” system and evaluated its benefits using ClinVar variants of at least two-star quality as use cases. The data of all human substitutions located in the protein domains are available on the DOLPHIN website: https://dolphin.mmg-gbit.eu.

## Material and methods

### Substitution scores for specific domains through positional score matrices

All protein domain information was extracted from the Pfam database (Finn et al., 2016). We used the Pfam 33.1 version of May 2020, containing 18,259 entries. Among these domains, we used the Pfam-A subset of 18,101 curated domains for further analysis (Sonnhammer et al., 1997). Each alignment was filtered to remove information from Archaea, bacteria, viruses, and other sequences to retain only data from eukaryotes. Overall, they contain information from 27,077,043 domains from 1,161 species. The human protein domains were extracted from the canonical Uniprot transcripts used in Pfam and represent 5,168,776 amino acids out of the 12,871,017 amino acids in human proteins (40.2%).

For each residue, we then calculated an amino acid value using the following steps: creation of a count matrix, a corrected frequency matrix, a corrected relative frequency matrix, and the position score matrix.

### The counting matrix

First, we created a script to count the occurrence of each amino acid at each position in the alignment and to transfer this data into a count matrix: C (p, l). Let A be a multiple alignment of length L and ψ a finite alphabet (single letter codes of amino acids). A count matrix denoted C representative of the alignment A is a matrix L × |ψ | such that for any letter l ∈ ψ and any position p ∈ {0 … L-1}, the index element l and p of C denoted C (p, l) is defined by the number of occurrences of the letter l in position p of the multiple alignment A.

### The corrected frequency matrix

From the count matrix, we created a corrected frequency matrix. The goal was to no longer observe a value equal to 0 by adding a pseudo-count to the starting frequency. Let ψ be a finite alphabet (single letter codes of amino acids), C a count matrix, l a letter ∈ ψ, p a position ∈ {0 … L-1}, c a pseudo-count and fl the expected frequency of the letter l. The corrected frequency matrix is denoted F′ and is defined by the following formula:

$$F^{\prime }\left(p,l\right)=\frac{C\left(p,l\right)+c\times f_{l}}{\sum_{l\in \psi }\;C\left(p,l\right)+c}$$

We arbitrarily chose a pseudo-count “c” equal to 1, this value being negligible compared to the values of the count matrices. Regarding the expected frequency of the “fl” amino acids, we used data from Uniprot/Swissprot (http://www.uniprot.org/statistics/Swiss-Prot%202013_04).

### The corrected relative frequency matrix

The purpose of this matrix was to re-contextualize the frequencies. Let ψ be a finite alphabet (single letter codes of amino acids), L a natural number, F′ a corrected frequency matrix, l a letter ∈ ψ, p a position ∈ {0 … L-1} and fl the expected frequency of the letter l with $\sum_{l\in \psi }f_{l}$ = 1. The corrected relative frequency matrix is denoted F″ and is defined by the following formula:

$$F^{\prime \prime }\;\left(p,l\right)=\frac{F^{\prime }\left(p,l\right)}{f_{l}}$$

### The score-position matrix

Finally, the corrected relative frequency matrix was modified to obtain the score-position matrix. For this, the logarithm of the corrected relative frequency was used. Let F″ be a corrected relative frequency matrix, ψ a finite alphabet (single letter codes of amino acids) and L be a natural number. A score-position matrix denoted M is a matrix L × |ψ | such that for any letter l ∈ ψ and any position p ∈ {0 … L-1}, the element of indices l and p of M denoted M (p, l) is defined by the following formula:

$$M\left(p,l\right)=\ln F^{\prime \prime }\left(p,l\right)$$

This matrix allowed us to move from a multiplicative to an additive model. Normally, this type of matrix determines when the frequency of the motif is greater than the frequency of the context (and *vice versa*). Here, we have slightly diverted this principle to calculate a “∆” score by subtracting the wild-type (wt) score from the mutant (mut) amino acid using the formula:

$$∆=M\left(p_{mut},l_{mut}\right)-M\left(p_{wt},l_{wt}\right)$$

### Frequency of a given substitution in a specific protein domain

All substitution frequencies (52,774,671 protein missense variants) were extracted from dbNSFP version 4.1a (16 June 2020), which provides information for all potential non-synonymous SNVs (and splice-site SNVs) in the human genome (Liu et al., 2020). We used the allele frequency column from gnomAD v2.1.1 (Koch, 2020) containing data from 125,748 exomes. For each missense variation in a specific domain, we selected the most frequent mutational event leading to that substitution in all proteins containing that domain. For example, the NP_000129.3:p.Thr2032Ser variant of the *FBN1* gene corresponds to the 135th residue of the Pfam “Calcium-binding EGF protein” (PF07645) domain. The DOLPHIN frequency of this specific residue was set to 2.41 × 10^−5^ extracted from the NP_001989.2:p.Thr784Ser variant of the *FBLN2* gene that impacts the 135th residue of this domain. Data were stored in a substitution frequency table.

### K-means clustering in R

To predict whether a substitution involves a key residue of a functional domain using DOLPHIN “wt” and “∆” scores, we used a commonly unsupervised machine learning algorithm: k-means clustering with R. The k-means clustering works by evaluating the distance between a point and the center of a cluster. Points are associated to the nearest k-center, then cluster centers of gravity are computed and become new centers. These steps are repeated over several iterations until stabilization is achieved. The optimal number of clusters is determined by computing k-means clustering using different k-clusters values and the WSS (Within Sum of Square) is drawn based on the number of clusters. The location of a kink in the plot indicates the appropriate number of clusters.

This approach was used on ClinVar variations extracted from the 28 July 2020 version of ClinVar (Landrum et al., 2016). All class 1, 2, 4 and 5 missense variants located in protein domains were selected and only those with a quality of at least two stars were used for further analysis. They contained 9,121 selected variants (4,382 pathogenic or probably pathogenic variants and 4,739 benign or probably benign variants).

### The DOLPHIN database and the website

The DOLPHIN database has been developed using PostgreSQL version 11 (postgresql.org). The web interface was created with the Laravel framework version 6 (“http://www.laravel.com”). The alignment logos were obtained from the Skylign tool (Wheeler et al., 2014). End users can query the system for a gene, transcript, or protein and then select a specific variant. Intuitive graphical displays and tables make it easy to retrieve HMM logos, alignments, predictions, as well as frequencies for all variants in protein domains (Supplementary Figure S1).

To allow rapid access to DOLPHIN data, an API is also available. Il allows retrieval of the gene symbol, variation using the HGVS p. nomenclature, the Pfam entry names, position of the variation in a protein domain, reference and alternative amino acids, DOLPHIN “wt”, “mutant” and ∆ scores, PM1 and PM2/BS1 predictions, as well as the domain symbol and name, from a single protein substitution localized in the protein domains (Supplementary Figure S2).

## Results

### Determination of thresholds for substitution classification using the DOLPHIN “wt” and “∆” scores

**FIGURE 1:**
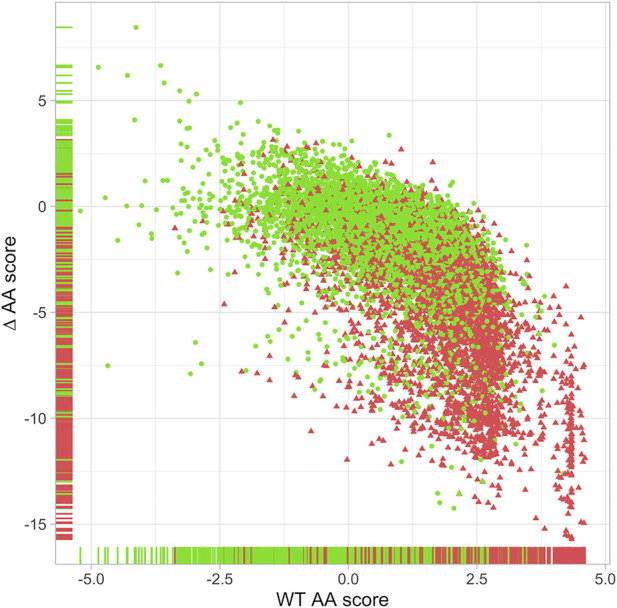
Distribution of the 9,121 ClinVar variations with a quality of at least two stars located in protein domains. *X*-axis = DOLPHIN “wt” scores; *Y*-axis = DOLPHIN “∆” scores. Red triangles = ClinVar Class 4 & 5 variations (*n* = 4,382). Green dots = ClinVar Class 1 & 2 variations (*n* = 4,739). Horizontal and vertical bands on *X* and *Y*-axis represent the corresponding “wt” and “∆” values of each variation according to their type: red for pathogenic variations (class 4 & 5) and green for neutral variations (class 1 & 2).

Using the “position score” matrices, we determined for each ClinVar variant (Landrum et al., 2016) its “wt” and “∆” scores, which are representative of a key residue and a significant amino acid change at that residue respectively (Figure 1). Combining these two pieces of information allowed us to define whether a specific substitution can be annotated with PM1 evidence representing a missense variation that significantly alters a key residue of a protein domain. A k-means clustering approach allowed us to distinguish 4 clusters, cluster A containing 92% of the pathogenic variants, cluster B 80%, and cluster C 45%, while cluster D contains 81% of the benign variants. These clusters were then individualized with threshold lines (Supplementary Figure S3). Cluster C is an area of overlapping pathogenic and benign variants, which does not allow for effective discrimination. Therefore, the DOLPHIN system only labels variants located in areas A and B as PM1.

### Comparison of DOLPHIN PM1 evidence with InterVar PM1 information

To assess the benefits of the new PM1 evidence provided by DOLPHIN, we compared the results with the InterVar system, which generates an automated classification of variant using 18 criteria including PM1 evidence (Li and Wang, 2017) (Supplementary Table S1).

If we make the assumption that pathogenic variants contained in protein domains have a high probability of impacting domain structure and/or function, we expect them to be annotated with PM1 evidence. As shown in Table 1, InterVar and DOLPHIN provide statistically different PM1 annotations, with an accuracy of 0.64 and 0.82, respectively. The main difference is in the rates of false positive (2,654 vs. 461) and true positives (3,707 vs. 2,379). InterVar false negatives correspond mainly to localized variations in protein domains that contain at least one benign variant as illustrated by the pathogenic variant NP_000426.2:p.Cys1061Tyr (*NOTCH3*). In other situations, they correspond to protein domains absent from Uniprot as illustrated by the pathogenic variant NP_000536.6:p.Pro447Leu (*HNF1A*) that is localized in the Pfam domain (PF04812) of HNF-1B that has no match in UniProt (Table 2, Supplementary Figures S4A, B1). InterVar false positives correspond to variants localized in protein domains without benign variations when the variant itself does not involve a key domain residue. This is illustrated by the benign variants NP_000242.1:p.Thr564Ala (*MSH2*) and NP_000359.1:p.Phe285Val (*TSC1*) (Table 2, Supplementary Figure S4C, D). DOLPHIN false negatives (e.g., pathogenic variants NP_714915.3:p.Leu349Ser (*TMEM67)* and NP_001186036.1:p.Arg242Cys (*TBC1D24)*) and false positives (e.g., benign variants NP_000240.1:p.Glu600Gly (*MLH1)* and NP_009225.1:p.Val1804Asp (*BRCA1)*) are all located in the C DOLPHIN zone, the area where pathogenic and benign variations overlap (Table 3, Supplementary Figure S5).

**TABLE 1:** PM1 classification of ClinVar variants localized in protein domains. TP = True Positives; TN = True Negatives; FP = False Positives; FN = False Negatives; SPE = Specificity; SEN = Sensibility; ACC = Accuracy; MCC = Matthews Correlation Coefficient.

	INTERVAR	DOLPHIN
NA	4	2,993
TP	3,707	2,379
TN	2,083	2,638
FP	2,654	461
FN	673	650
SPE	0.44	0.85
SEN	0.85	0.79
ACC	0.64	0.82
MCC	0.31	0.64

**TABLE 2:** False negatives and false positives of the InterVar PM1 annotation based on InterVar, DOLPHIN and Varsome. AA = Amino Acid.

Variants	Clinvar classification	Systems	PM1	Details	Supp.figure	Area
*NOTCH3* NP_000426.2:p.Cys1061Tyr	Pathogenic/Likely pathogenic	DOLPHIN	Yes	Domain: EGF (PF00008) AA #1051 to 1080, wt = 4.3, ∆ = -10.8	4A	A
		InterVar	No	—		
		VarSome	No	Uniprot protein “EGF-like 27” has 6 non-VUS missense/in-frame/non-synonymous variants (5 pathogenic and 1 benign) = 83.3%, which is more than threshold 50.0%		
*HNF1A* NP_000536.6:p.Pro447Leu	Pathogenic	DOLPHIN	Yes	Domain: HNF-1B (PF04812)	4B	C
				AA #282 to 540, wt = 2.9, ∆ = -9.3		
		InterVar	No	—		
		VarSome	No	No domain in Uniprot		
				Hotspot of length 17 amino-acids has only 1 pathogenic missense/in-frame variants, which is less than minimum of 6		
*MSH2* NP_000242.1:p.Thr564Ala	Benign	DOLPHIN	No	Domain: MutS_IV (PF05190)	4C	D
				AA #473 to 569, wt = 0.03, ∆ = -0.4		
		InterVar	Yes	—		
		VarSome	No	No domain in Uniprot		
				Hotspot of length 17 amino-acids has 4 pathogenic missense/in-frame variants, which is less than minimum of 6		
*TSC1* NP_000359.1:p.Phe285Val	Benign/Likely benign	DOLPHIN	No	Domain: Hamartin (PF04388)	4D	D
				AA #7 to 719, wt = 1.3, ∆ = -2.3		
		InterVar	Yes	—		
		VarSome	No	No domain in Uniprot		
				Hotspot of length 17 amino-acids has 0 pathogenic missense/in-frame variants, which is less than minimum of 6		

**TABLE 3:** False negatives and false positives of the DOLPHIN PM1 annotation based on DOLPHIN, InterVar and Varsome. AA = Amino Acid; UK = Unknown.

Variants	ClinVar classification	System	PM1	Details	Supp. figure	Area
***TMEM67*** NP_714915.3:p.Leu349Ser	Pathogenic/Likely pathogenic	DOLPHIN	UK	Domain: Meckelin (PF09773)	5A	C
				AA #167 to 995, wt = 1.2, ∆ = -2.4		
		InterVar	Yes	—		
		VarSome	No	No domain in Uniprot		
				Hotspot of length 17 amino-acids has only 1 pathogenic missense/in-frame variants, which is less than minimum of 6		
***TBC1D24*** NP_001186036.1:p.Arg242Cys	Pathogenic/Likely pathogenic	DOLPHIN	UK	Domain: RabGAP-TBC (PF00566)	5B	C
				AA #48 to 255, wt = 1.5, ∆ = -0.7		
		InterVar	Yes	—		
		VarSome	Yes	UniProt protein TBC24_HUMAN domain “Rab-GAP TBC” has 15 non-VUS missense/in-frame/non-synonymous variants (14 pathogenic and 1 benign), pathogenicity = 93.3% which is more than threshold 50.0%		
***MLH1*** NP_000240.1:p.Glu600Gly	Benign	DOLPHIN	UK	Domain: Mlh1_C (PF16413)	5C	C
				AA #502 to 756, wt = 1.4, ∆ = -2.8		
		InterVar	No	—		
		VarSome	Yes	UniProt protein MLH1_HUMAN region of interest ‘Interaction with EXO1’ has 66 non-VUS missense/in-frame/non-synonymous variants (50 pathogenic and 16 benign), pathogenicity = 75.8% which is more than threshold 50.0%		
***BRCA1*** NP_009225.1:p.Val1804Asp	Benign	DOLPHIN	UK	Domain: BRCT (PF00533)	5D	C
				AA #1756 to 1842, wt = 1.3, ∆ = -4.6		
		InterVar	No	—		
		VarSome	Yes	UniProt protein BRCA1_HUMAN domain “BRCT 2"has 64 non-VUS missense/in-frame/non-synonymous variants (37 pathogenic and 27 benign), pathogenicity = 57.8% which is more than threshold 50.0%		

Since the ClinVar classification takes into account all ACMG criteria, it is very likely that some missense variations classified as pathogenic may indeed impact on splicing and thus appear as false negatives when considering DOLPHIN scores. To evaluate this hypothesis, we used the HSF Pro system from GenOmnis (genomnis.com) (Desmet et al., 2009) on all false-negative variants located in zones C and D. We found that among the 2,003 variants, 61 have an impact on splicing (out of a total of 100 in the entire dataset), 314 have a potential impact, 820 probably have no impact and finally 808 have no impact (Supplementary Figure S6).

### Frequency of reported variations in protein domains

The 18,101 unique domains extracted from the Pfam-A subset contain information from 105,178 domains from the human genome (6,533 unique domains). In total, these domains contain 5,168,776 amino acids out of the 12,871,017, which represents appoximately 40.2% of the human proteins when considering the UniProt entry name used by Pfam. Of the 52,774,671 potential substitutions extracted from dbNSFP 4.1a (Liu et al., 2016) in protein domains, 3,990,680 are annotated in gnomAD (Koch, 2020) (7.6%). DOLPHIN provides extrapolated frequency information for 16,764,498 amino acid substitutions (31.8%).

**FIGURE 2:**
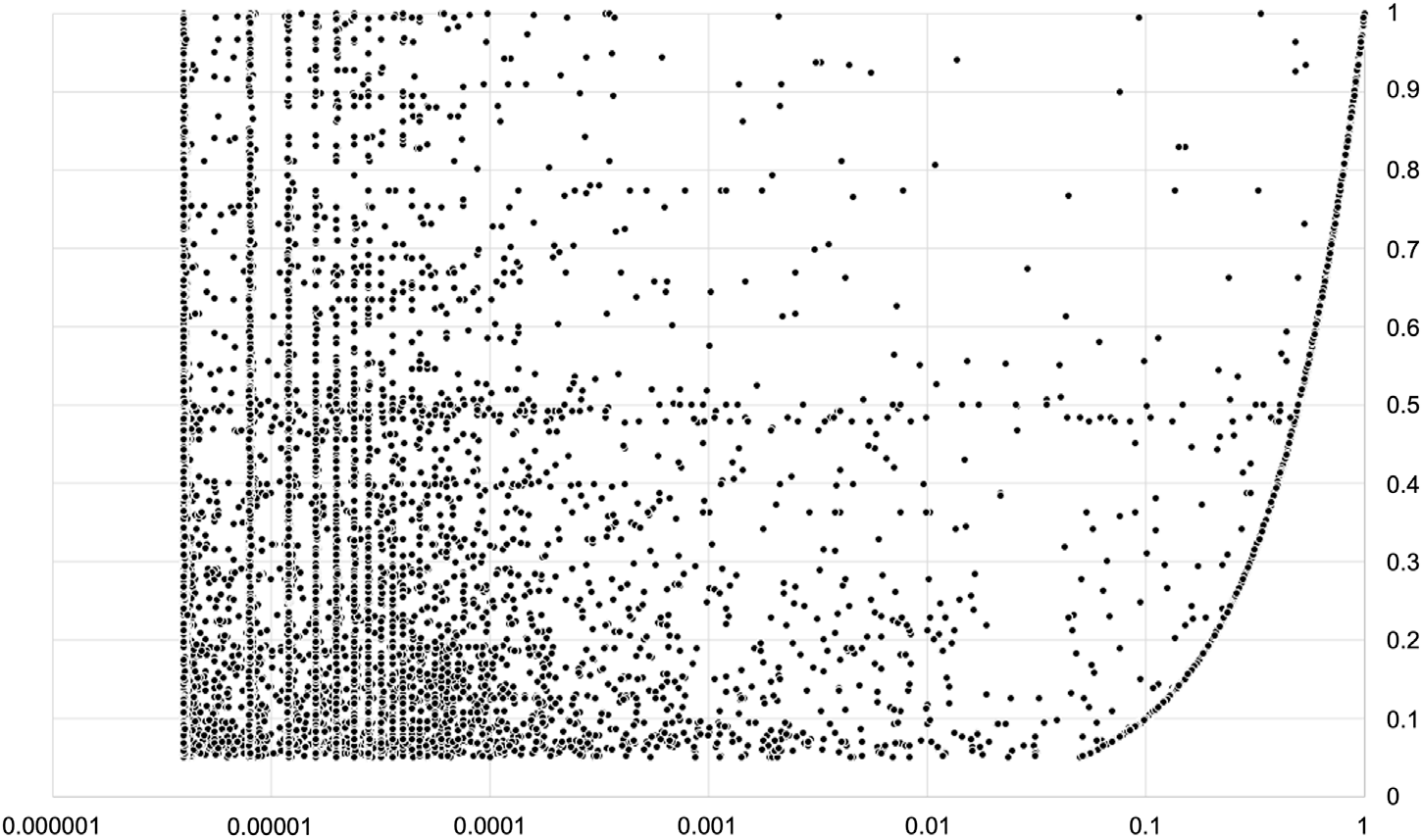
Variations localized in protein domains with a DOLPHIN frequency greater then 5% and reported in gnomAD. *X*-axis (logarithmic scale) = gnomAD frequency; *Y*-axis = DOLPHIN Frequency. Note that only a minority of variations (2.4%) have the same frequency in both datasets.

Among the DOLPHIN annotations, we extracted two groups of variants: A) those with a frequency ≥5% and, B) those with frequency between 1% and 5% that are highly relevant as annotated as BA1 or BS1 ACMG classification evidence. We found 398,443 and 228,844 of these variants, respectively. 386,934 (97.1%) and 221,073 (96.6%) respectively were reported with lower gnomAD frequencies, as shown in Figure 2.

Of group A, 647 were previously reported in ClinVar (0.16%). Six hundred thirty-seven were annotated as Benign or Likely Benign (98.5%). Of these, 614 were previously reported in gnomAD with a MAF ≥0.05, whereas 22 were reported with an average frequency of 0.57% ± 1% (1.5 10^−2^–4.8%). Three of the remaining 4 variants were reported in ClinVar as Likely Pathogenic or Pathogenic (1.1%). Of group B, 489 were previously reported in ClinVar (0.21%), of which 481 were reported as benign or likely benign (98.4%).

### Protein domain information from DOLPHIN in relation to protein conservation

Many *in silico* pathogenicity prediction tools use protein conservation information extracted from the conservation of 100 species published by UCSC using phyloP (Pollard et al., 2009) and phastCons (Siepel et al., 2005) based on the “multiz100way” multiple sequence alignment (MSA) generated by Multiz software (Blanchette et al., 2004). To assess whether amino acid conservation of a given protein in different species provides distinct information about the conservation of these amino acids in protein domains, we used the 9,121 ClinVar variants. As shown in Supplementary Figure S7, using the Spearman rank correlation coefficient, we obtained a moderate positive correlation (ρ = 0.50). This difference is illustrated by the uneven distribution of conservation, with 54% of residues showing very high conservation between species (>85%) *versus* only 3.6% showing very high conservation within domains.

This difference can be illustrated by the example of the 37th (Supplementary Figure S8A) and 10th (Supplementary Figure S8B) repeated “calcium binding EGF-like domain” (PF07645) of the fibrillin-1 protein, which contains 37 such domains in Pfam.

## Discussion

The evolution of the gene-coding portion of eukaryotic genomes has resulted in multiple proteins, many of which share protein domains (Bagowski et al., 2010). By definition, these domains are characterized as conserved, functionally independent protein sequences that bind or process ligands using a central structural motif (Bagowski et al., 2010). Each domain shares common functions and structures within various proteins and species. Throughout evolution, variations have affected these elements and have been subject to genetic drift or selection. Studying domains from multiple species provides a new layer of information that can be used to facilitate the classification of human variants. It takes advantage of the variety of variation rates that exist in different organisms due to their population number, generation turnover, dissimilar metabolisms, interaction with the environment, and reproductive strategies, allowing conclusion to be drawn about the evolution of protein domain over much longer periods of time than just human evolution. We therefore built the DOLPHIN system not only to annotate residues in key protein domains but also to refine substitution frequencies in the human population. Both of these are essential to the ACMG-AMP guidelines for classification of variants in research and diagnostics of rare human genetic diseases or cancers (Richards et al., 2008; Richards et al., 2015) through the PM1 and PM2/BS1 criteria.

Various resources have been developed to use protein domain information for variant classification, such as the MetaDome (Wiel et al., 2017; Wiel et al., 2019) or the Prot2HG systems (Stanek et al., 2020). Both use only human variations and do not benefit from all information available in eukaryotes like DOLPHIN. The importance of protein domain information has also been explored for classifying somatic mutations through the “domain-centric” approach used in OncoDomain (Peterson et al., 2017) that takes into account somatic variants from The Cancer Genome Atlas (TCGA). We believe that the selection pressure is different when considering somatic events in cancer cells *versus* germline events and their impact on the whole organism. DOLPHIN and OncoDomain are therefore complementary.

Today, automatic classifiers, such as InterVar and VarSome, do not assign the PM1 criterion in the same way, even though they both use data from the same reference databases (e.g., InterPro). Thus, InterVar excludes all protein domains containing variants annotated as benign or common (allele frequency >5%) and does not consider hotspots. VarSome considers protein domains that contain at least one annotated pathogenic variant if the ratio of pathogenic variants *versus* non-pathogenic and VUS variants is above 50%. In addition, both approaches rely on manual annotation of variants, which introduces a bias because only a minority of the observed variants are currently classified. In comparison, DOLPHIN, using only residues conservation in eukaryotes, is free from annotation of variants and the presence/absence of benign variants. To do so, it uses “wt” and “∆” scores for each variant located in a protein domain. Based on the variants in the ClinVar database located in protein domains and a k-means clustering approach, we demonstrated that DOLPHIN could efficiently assign a PM1 label. This was extended to all potential variants in human transcripts, resulting in 15,841,959 variants (30.0%) being assigned the PM1 label. We therefore propose to restrict the PM1 criterion to this subset of variants that was determined by a standardized approach provided by DOLPHIN, consistent with the harmonization goals of the ACMG-AMP recommendations.

We believe that the information associated with mutational hotspots should be treated separately. Indeed, these elements are, on the one hand, independent of the protein domains and, on the other hand, provide only indirect information on the variant by answering the question: is it located in a small region where many pathogenic variants are present? This information could then be proposed with a lower weight (“support”)?

In addition to using DOLPHIN scores to label a PM1 variant, this data can also be used to label a variant as having no significant impact on a protein residue. We therefore propose to create a BP8 criterion “Located in a functional domain without affecting a key residue”. This new label could be assigned to 17,523,715 variants (33.2%) using the same standardized approach *via* DOLPHIN. In total, DOLPHIN will provide a PM1 or BP8 label to 63.2% of variations localized in protein domains. Arguably, BP8 is a computational criterion that can overlap the BP4 criterion provided by *in silico* tools. We believe that BP8 should not be considered as computational evidence but rather as observational evidence.

Concomitantly, as Amendola et al. (2016) report, the most widely used criteria for variant classification are PM2/BA1/BS1 “variant absent in population databases or with an allele frequency too high for the disease”. As more and more genomes are sequenced and their data shared in central databases as gnomAD (Koch, 2020), their variant frequency content is directly linked to human evolution in relation to genetic drift and selection pressure. Thus, even if tens of millions of samples were available, these evolutionary forces could limit this information. One way to circumvent this limitation is to access another layer of information that is provided by the protein domains. Because these domains are present in multiple proteins, they represent a much longer evolutionary period for a larger population and provide much more information. Comparing of the gnomAD and DOLPHIN data reveals that the former provides information for 3,990,680 (7.6%) residues out of the 52,774,671 potential protein domains substitutions, while the latter provides frequencies for 16,764,498 amino acid substitutions (31.8%).

We are fully aware that the frequency information in DOLPHIN does not give the exact frequency of a given amino acid substitution at a given protein residue, unlike gnomAD, but rather the highest frequency of that specific substitution among all corresponding protein domains in any human protein. To demonstrate that this new information is valuable, we selected all substitutions whose frequency in DOLPHIN is greater than 5% (398,443 variations), theoretically corresponding to “BA1 evidence” ACMG/AMP and those whose frequency is between 1% and 5% (386,934) corresponding to “BS1 evidence”. Respectively, 98.5% and 98.4% of those reported in ClinVar were classified as benign or probably benign, demonstrating that DOLPHIN frequencies above 1% are strongly associate with benign variations.

Because the DOLPHIN frequency is an extrapolation of the true frequency of a variant, we propose that this information, when it is greater than or equal to 1%, be used as a BS1 criterion. When it is null (a variant has never been observed in any of the corresponding protein domains), it could then be annotated with the PM2 criterion. This annotation has, according to us, a higher specificity than gnomAD since only 63.2% of the variants contained in DOLPHIN have this label *versus* 92.4% with GnomAD.

Despite the standardized approach used in DOLPHIN, some limitations remain. For example, it is not possible to annotate as PM1 or BP8 the ambiguous variants located in the C-zone. Futhermore, like most systems using protein-level information, DOLPHIN may incorrectly label substitutions that impact the mRNA level. This led us to propose to classify as BS1 only variants with a high DOLPHIN frequency (>5%) that should theoretically be classified as BA1. Indeed, this “protein” frequency can be derived from a different nucleotide context than the original variant.

In conclusion, we developed a new system called DOLPHIN to extract information from protein domains. These data are freely available from the DOLPHIN website at https://dolphin.mmg-gbit.eu, in accordance with Open Science recommendations (https://en.unesco.org/science-sustainable-future/open-science/recommendation). We were able to demonstrate that this system allows a re-evaluation of the PM1 and PM2/BS1 criteria and the creation of a new BP8 classification criterion by a standardized approach in agreement with the objectives of harmonization of the ACMG-AMP recommendations. We believe that it allows an easier classification of amino acid substitutions in protein domains that represent nearly 40.2% of proteins and constitute the sites of most pathogenic mutations (Iqbal et al., 2020).

## Data Availability

The datasets presented in this study can be found in online repositories. The names of the repository/repositories and accession number(s) can be found below: https://dolphin.mmg-gbit.eu.
