# Supplementary material for: Protein domains provide a new layer of information for classifying human variations in rare diseases
Source: Front Bioinform. 2023 Feb 21;3:1127341. doi: 10.3389/fbinf.2023.1127341 (PMC9990413; doi:10.3389/fbinf.2023.1127341)
Supplement: Supplementary file 1 [file Table2.DOCX]

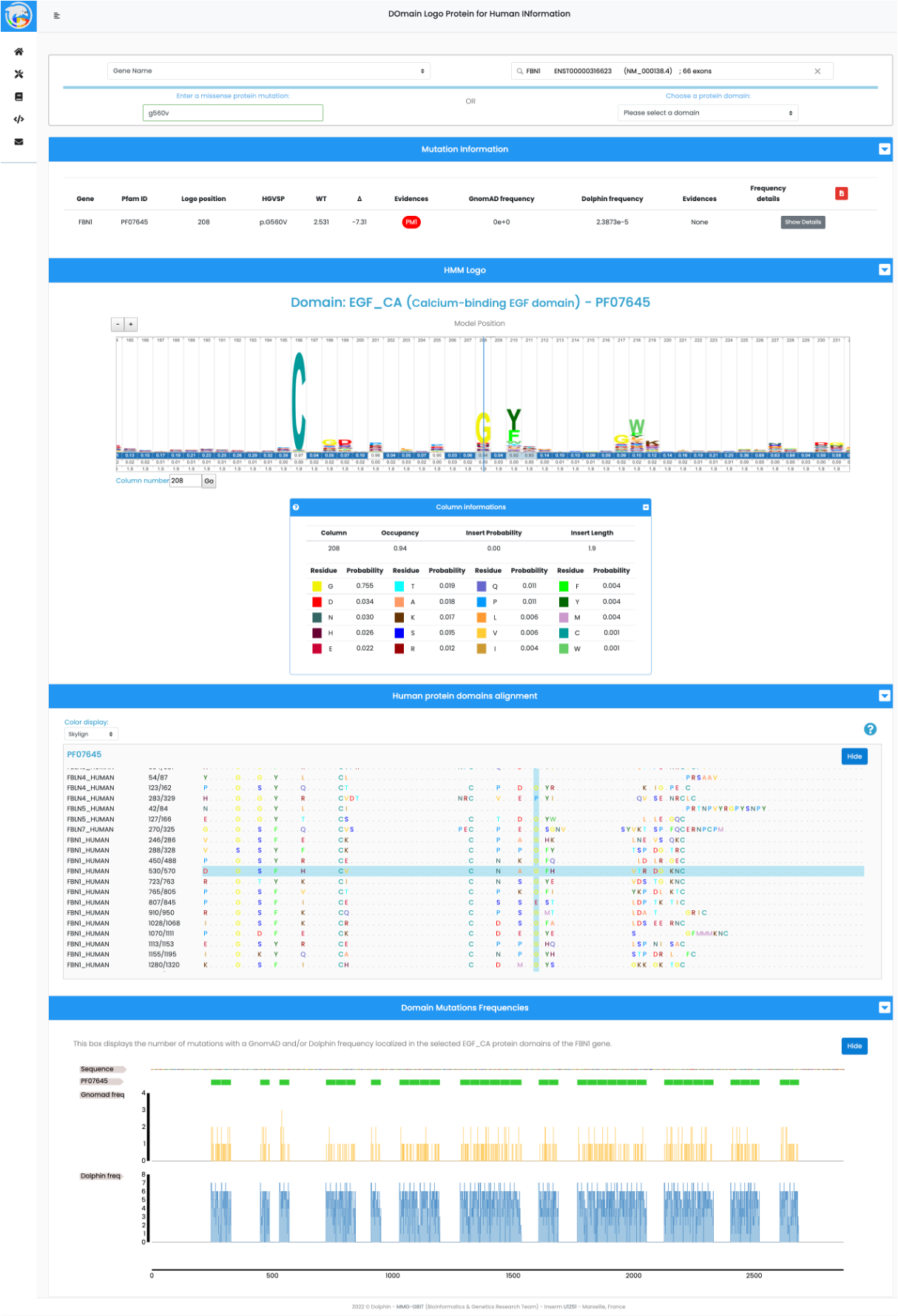


**Supplementary Figure 1:** **Display of the NP_000129.3:p.Gly560Val variation from the *FBN1* gene (ENST00000316623) from the DOLPHIN website.** Top: Query box. HMM Logo box: display of the full HMM domain logo containing the selected variation. DOLPHIN scores, prediction and gnomAD and DOLPHIN frequencies are displayed here and can be copied. Human Domain Protein Alignment bow: contains the alignment of the domain in all corresponding human proteins. The color code can be customized using the Skylign, default, clustalX, Rasmol and Rasmol shapely color scheme. Domain Variations Frequencies box: contains the corresponding protein domains from the selected gene/transcript (green boxes). The yellow and blue bar plots display the gnomAD and DOLPHIN number of substitutions with a frequency at each protein positions within domains.


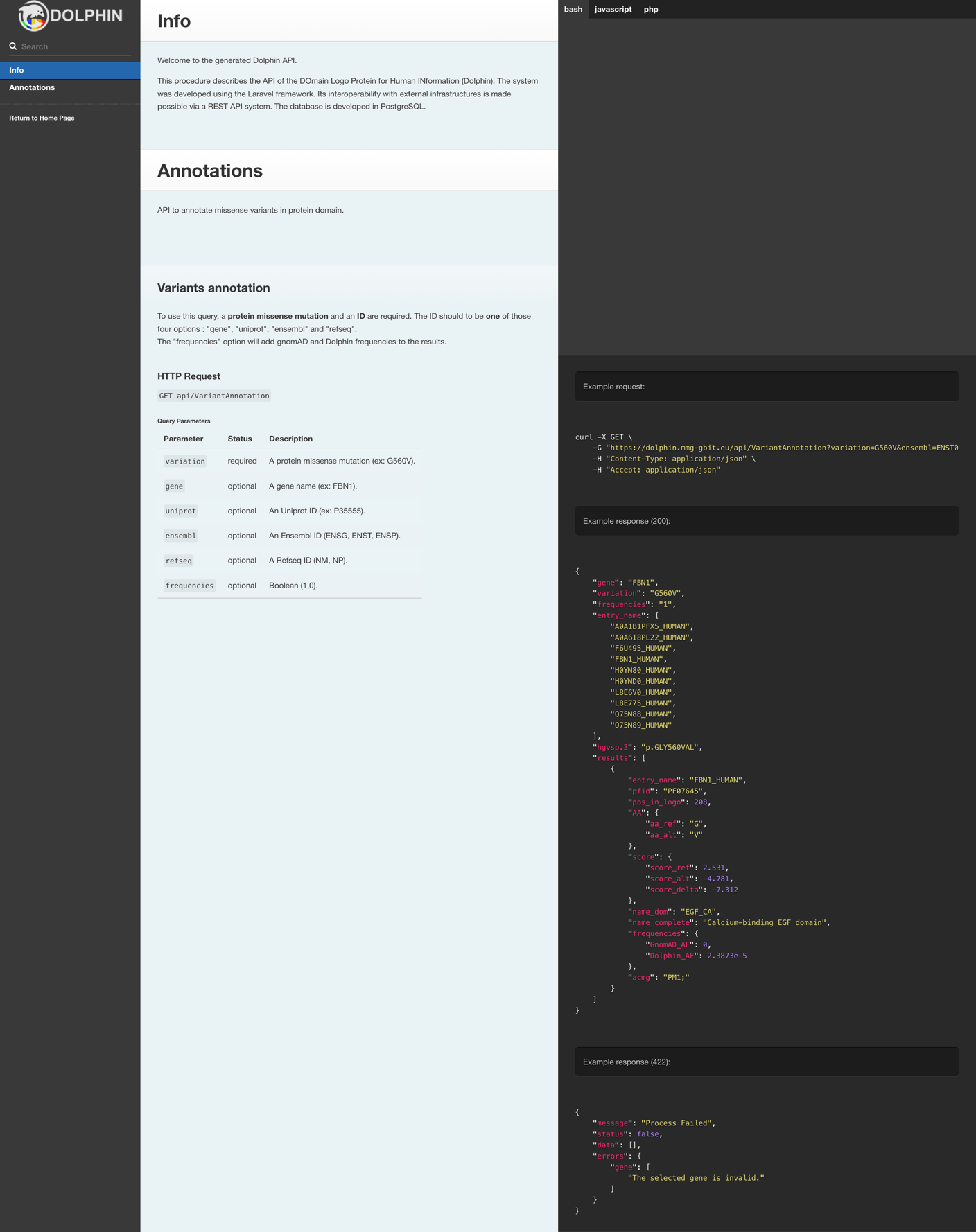


**Supplementary Figure 2:** **Result of the DOLPHIN API** for the p.Gly560Val variant from the *FBN1* gene. Information from all transcripts is included if the corresponding variation is localized in a protein domain.


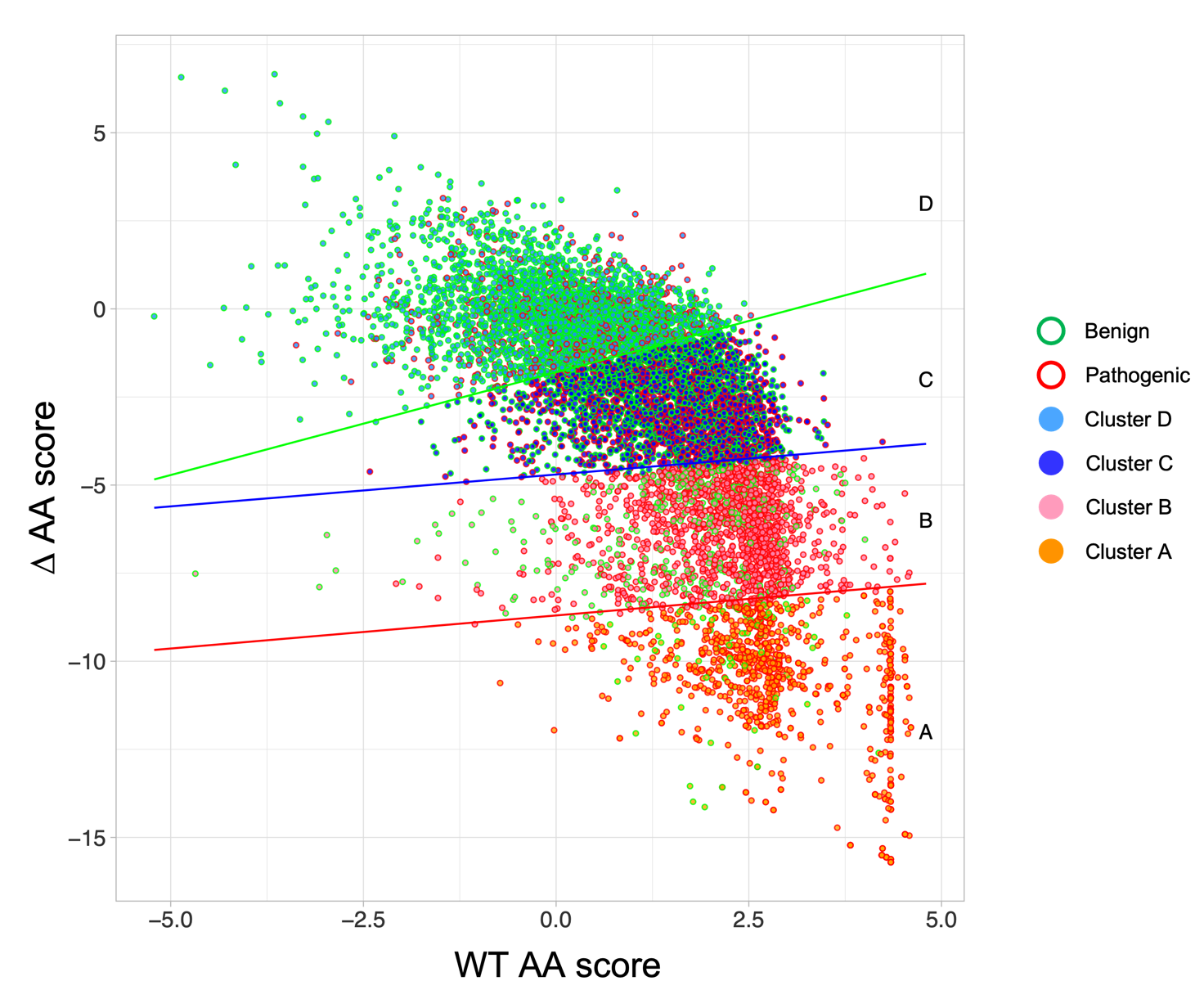


**Supplementary Figure 3:** **Distribution of 9,121 ClinVar variants** **with at least a 2-stars quality localized in protein domains**. X-axis = DOLPHIN “wt” score; Y-axis = DOLPHIN “∆” score. Circles with red border = ClinVar Class 4 & 5 variants (n = 4,382). Circles with green border = ClinVar Class 1 & 2 variants (n = 4,739). Red outline = Pathogenic variants; Green outline = Benign variants. Orange = variants localized in cluster A that contains 92% of pathogenic variants; Pink = variants localized in cluster B that contains 80% of pathogenic variants; Light blue = variants localized in cluster C that contains 45% of pathogenic variants; Dark blue = variants localized in cluster D that contains 81% of benign variants.


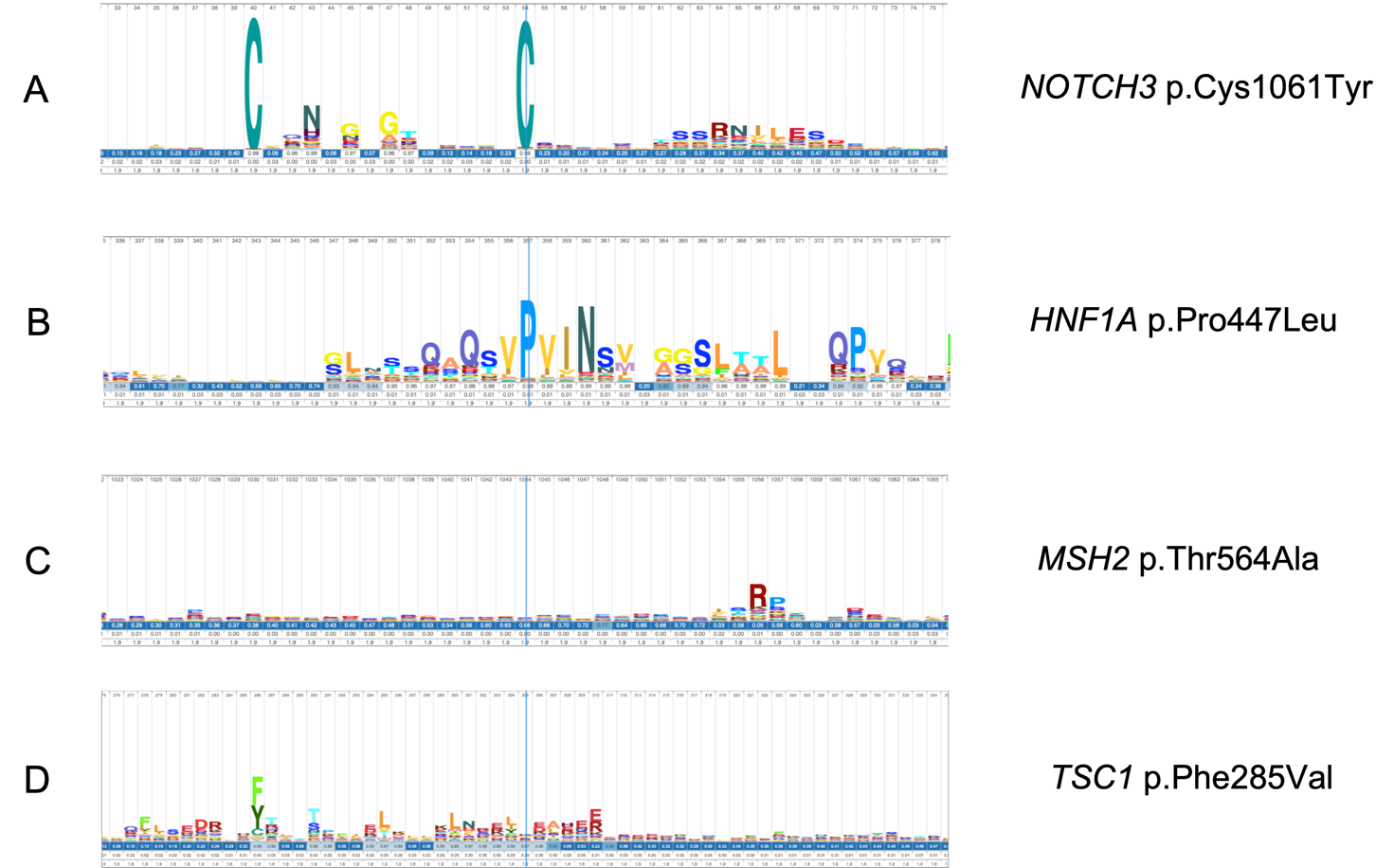


**Supplementary Figure 4: HMM Logo of InterVar false negative and false positive variant examples.** HMM Logos were generated by the Skylign tool and are available on the DOPLHIN’s website. A tall letter indicates a highly conserved amino acid in the protein domain. The relevant amino acid is centered and hovered over by the central blue line. **A**: (*NOTCH3*) NP_000426.2:p.Cys1061Tyr; **B**: (*HNF1A*) NP_000536.6:p.Pro447Leu; **C**: (*MSH2*) NP_000242.1:p.Thr564Ala; **D**: (*TSC1*) NP_000359.1:p.Phe285Val.


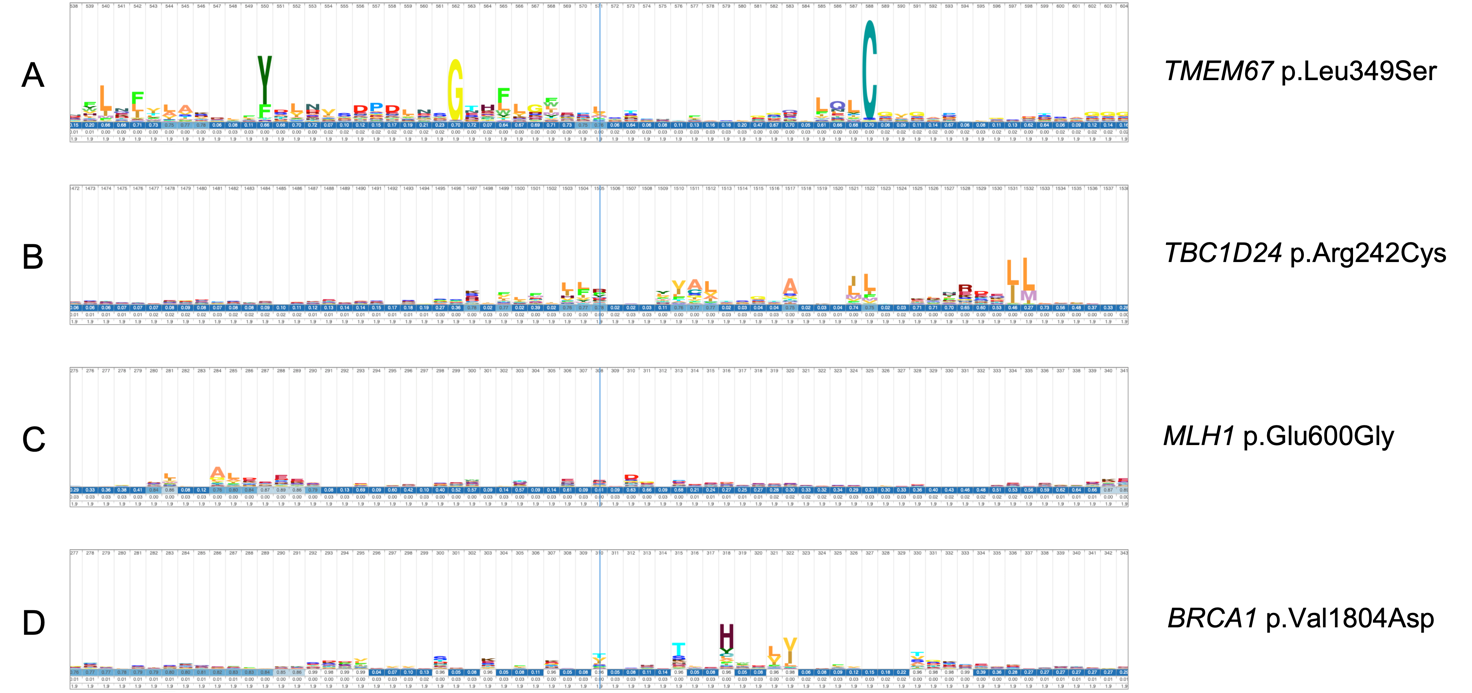


**Supplementary Figure 5: HMM Logo of DOLPHIN false negative and false positive variant examples.** HMM Logos were generated by the Skylign tool and are available on the DOPLHIN’s website. A tall letter indicates a highly conserved amino acid in the protein domain. **A**: (*TMEM67*) NP_714915.3:p.Leu349Ser; **B**: (*TBC1D24*) NP_001186036.1:p.Arg242Cys; **C**: (*MLH1*) NP_000240.1:p.Glu600Gly ; **D**: (*BRCA1*) NP_009225.1:p.Val1804Asp.


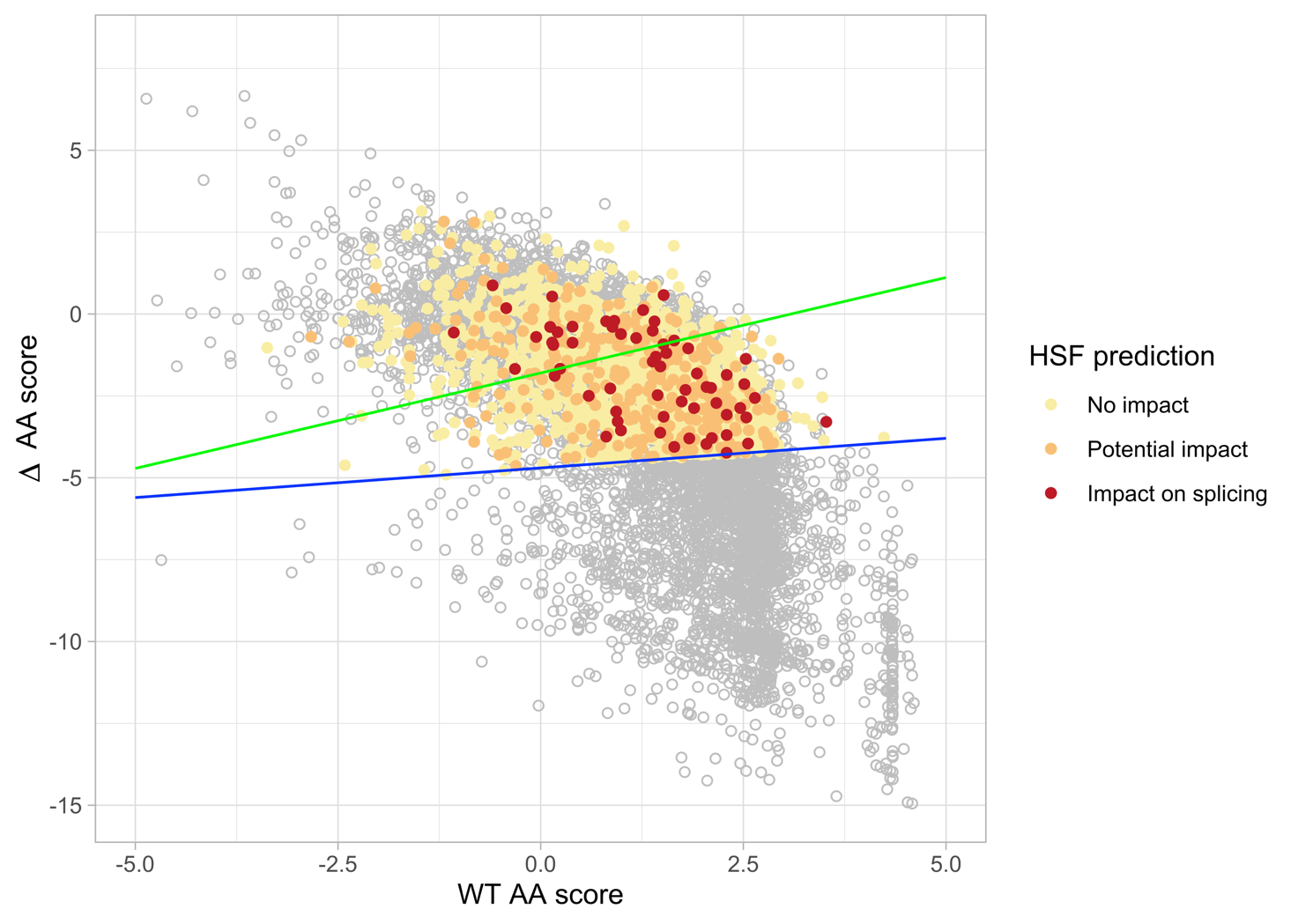


**Supplementary Figure 6: Human Splicing Finder prediction on DOLPHIN’s false negatives in C and D areas.** X-axis = DOLPHIN “wt” scores; Y-axis = DOLPHIN “∆” scores. Yellow dots = No impact on splicing; Orange dots = Potential impact on splicing; Red dots = Impact on splicing. D area above blue line; C area between blue and purple lines.

**
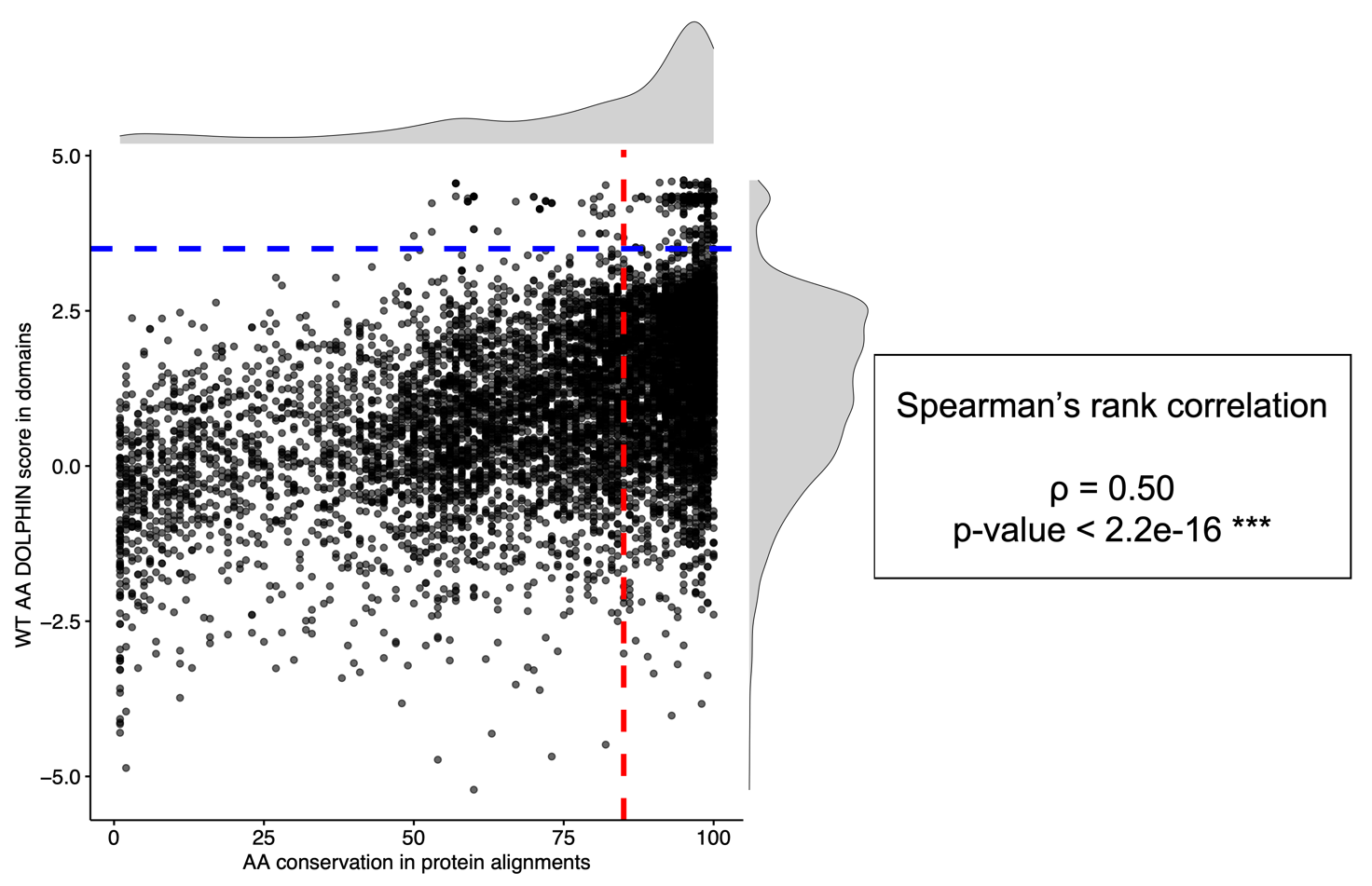
**

**Supplementary Figure 7: Correlation between protein domains information and protein conservation.** We used the Spearman rank correlation coefficient. X-axis = Amino acid conservation in protein alignment (multiz100way); Y-axis = DOLPHIN “WT” score. Red line = 85% conservation threshold. Blue line = 3.5 WT score threshold.

**
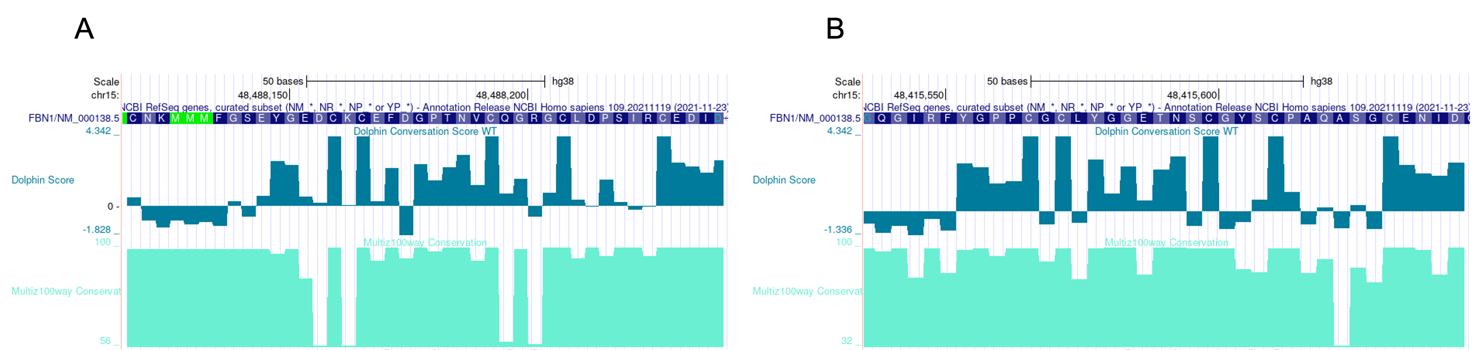
**

**Supplementary Figure 8: Comparison of protein and domain conservation information.** Top = Genomic reference sequence (HG38). NCBI RefSeq gene track of the amino acid sequence from the fibrillin-1 protein (*FBN1*). Dolphin score track (blue) = DOLPHIN amino-acid wild-type score. Multiz100way conservation track (green) = UCSC multiz alignment (exons translated) amino-acid conservation.

**A**: Calcium Binding Domain #10 (Pfam) of the fibrillin-1. **B**: Calcium Binding Domain #37 (Pfam) of the fibrillin-1.
